# End-Use Quality of Historical and Modern Winter Wheats Adapted to the Great Plains of the United States

**DOI:** 10.3390/foods11192975

**Published:** 2022-09-23

**Authors:** Sujun Liu, Lan Xu, Yifan Wu, Senay Simsek, Devin J. Rose

**Affiliations:** 1 Department of Food Science and Technology, University of Nebraska, Lincoln, NE 68501, USA; 2 Department of Agronomy & Horticulture, University of Nebraska, Lincoln, NE 68501, USA; 3 Whistler Center for Carbohydrate Research, Department of Food Science, Purdue University, West Lafayette, IN 47907, USA

**Keywords:** Mixograph, baking, whole grain, wheat flour, kernel quality

## Abstract

Improving milling and baking properties is important during wheat breeding. To determine changes in milling and baking quality of hard winter wheat, 23 adapted cultivars released in the Great Plains between 1870 and 2013 were grown in triplicate in a single location (Mead, NE, USA) over two crop years (2018 and 2019). Grain yield and kernel hardness index increased by release year (*p* < 0.05). The observed increase in hardness index was accompanied by a decrease in percent soft kernels (*p* < 0.05). Diameter and weight decreased with release year in 2019 (*p* < 0.05), and their standard deviation increased with the release year (*p* < 0.05). Flour protein content decreased with release year (*p* < 0.05) and dough mixing quality increased (*p* < 0.05). No significant relationship was found for baking property variables, but bran water retention capacity (BWRC), which is correlated with whole wheat bread quality, increased with release year (*p* < 0.05). In conclusion, wheat kernels have become harder but more variable in shape over a century of breeding. Mixing quality showed significant improvements, and loaf volume and firmness remained constant, even in the presence of a decrease in protein concentration. Bran quality decreased across release year, which may have implications for whole grain baking quality and milling productivity.

## 1. Introduction

For nearly a century, modern breeding efforts have been applied to wheat to improve yield, disease resistance, end-use quality, and other factors [1,2,3,4,5,6,7,8]. Although buffered by the polyploid nature of wheat, modern wheat has changed considerably from its ancestors. For example, modern wheat has improved in spike shattering, wind-scattering, and harvesting compared to historical cultivars [9].

Many studies show there is a relationship between wheat kernel physical properties and milling quality. For instance, the most important milling property is milling yield. A recent study about the effect of kernel size on milling yield and baking quality reported that small kernels contribute to enhanced bread-making quality but have a negative effect on milling yield [10]. Sutton et al., also showed that as wheat kernel size increased, flour yield increased [11]. Kernel size uniformity is also critical in the wheat milling industry since it is difficult to identify the best machine operating parameters when kernel size is not consistent. In the presence of large kernels, small kernels pass through the roller mills unground or merely partially broken [12,13]. Kernel hardness can also influence the milling process. For example, harder kernels require more energy to mill [13]. Similar to kernel size, many studies suggested that uniformity of kernel hardness is desirable for good milling performance [14,15].

Bran friability and bran water retention capacity (BWRC) are two new wheat quality measurements used to assess the potential quality of whole wheat flour. To assess bran friability, the proportion of bran retained on a no. 20 sieve relative to that retained on a no. 60 sieve is measured and termed “bran-friability”. Higher bran friability indicates lower whole wheat bread-specific volume [16]. BWRC is the weight of water retained by bran after centrifugation and is negatively correlated with whole grain baking quality [17].

Paramount among end-use quality traits of hard wheats is bread-making quality. Baking quality includes dough mixing properties and bread loaf quality and depends heavily on wheat protein quality and wheat protein content. Protein quality relates to the composition of glutenin and gliadins, the relative proportions of different protein classes, and the molecular weight distribution of the glutenin polymers. It will affect the loaf volume, shape ratio, crumb, and crust structure. Protein quality can be measured using protein size distribution measurements and mixers designed to measure the rheological properties of wheat dough [18].

During the wheat breeding process, milling, and baking properties are analyzed and considered important for breeding decisions. Several studies have examined how breeding has affected quality parameters, including kernel characteristics and flour solvent retention capacities, protein concentration and functionality, and mixing and baking properties over time [1,2,3,4,5,6,7,8]. For example, compared with historical wheats, modern wheats have improved grain yield and physical dough quality and stability [3,5,7]. Kernel quality and chemical composition have been compared between ancient and modern wheats. It was reported that historical cultivars are very different compared with modern hard red spring wheat, with modern wheat being harder and heavier in test weight [19]. Several findings suggest that the bread baking quality has improved successfully over the last century [3,8,20,21]. Flour from historical wheats has been shown to have higher concentrations of protein but lower glutenin, which is the major gluten protein fraction that is a suitable predictor for baking volume. Doughs from historical wheats can reach the maximum viscosity quickly and are characterized by low consistency and poor resistance to over-mixing [22]. Konvalina et al., performed an experiment on the baking quality of historical and modern wheat varieties [8]. They found that historical wheat cultivars are a valuable material with high protein content but may be more suitable for non-yeast-leavened products, such as pasta, biscuits, etc., than bread. Milling, rheological and bread-making performances of six historical wheat varieties have been investigated and compared with common Italian wheat. Compared with modern wheat, the bread made with historical wheat exhibited an overall lower specific volume and lower firmness [20]. In Canadian western red spring wheat, kernel weight, grain protein concentration, sodium dodecyl sulfate sedimentation volume, farinograph absorption, and dough development time all rose over time, indicating improvements in key agronomic and end-use traits over time [1].

While these studies report numerous significant improvements in the functionality of modern wheat compared with historical cultivars, there is little information on the changes that have occurred in winter wheats adapted to the Great Plains of the US. As previous studies have shown varying effects on quality depending on wheat class or growing region [1,2,3,4,5,7], this study aimed to assess the quality of winter wheat cultivars adapted to this region. We analyzed the most important kernel physical characteristics, milling, mixing, and baking qualities to determine how wheat changed during breeding and how the different quality variables were associated. We also analyzed two new wheat quality parameters that are associated with whole grain baking quality: Bran water absorption capacity (BWRC) and bran friability.

## 2. Materials and Methods

### 2.1. Materials

Twenty-three hard winter wheat cultivars, including two landraces and twenty-one elite cultivars released in the US between 1870 and 2013, were grown at the University of Nebraska Eastern Nebraska Research and Extension Center (ENREC) near Mead, NE, USA, and harvested in 2018 and 2019 (Table 1). The cultivars used in this study originated from the US states of Kansas, Nebraska, Texas, and Oklahoma and ranged in release years from 1870 to 2013. They were selected based on their relevance for grain production during their time, known adaptation to the climatic conditions of the study location, and their contribution to the pedigrees of modern genotypes widely grown in the Great Plains today. Before sowing, 1 m^2^ plots were prepared by applying a nitrogen fertilizer (90 kg/ha), as is standard practice in the region. Materials were planted in a randomized complete block design with three replications (field replicates) with the exceptions of ‘Anton’ with 4 replications and ‘Wesley’ with 2 replications in the 2019 harvest year (2018 planting). For each cultivar, plant height was measured in cm from the ground to the tip of spike, excluding awns at maturity. To determine grain yield, seeds from each plot were harvested using a simple plot combine harvester.

**Figure 1:**
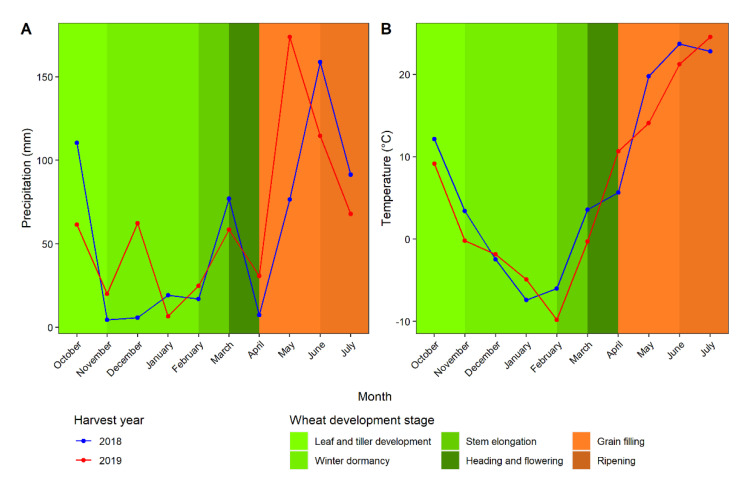
Precipitation (**A**) and temperature (**B**) condition and wheat development at Mead, NE, USA in 2018 and 2019.

Weather data, including precipitation and temperature for the growing location, was obtained from the High Plains Regional Climate Center [23]. The environmental conditions varied between harvest years (Figure 1). In 2019, conditions were not as favorable for wheat production due to the cold winter (with little snow cover). Additionally, ample rain early in the spring gave way to excessive dryness during grain filling. This may explain some of the differing trends across release years between the two planting years, as described in the results.

**Table 1:** Release year, origin, plant introduction (PI) or cereal introduction (CI) number, and reduced height (Rht-B1/RhtD1) genes for the wheat cultivars used in this study ^a^.

Cultivar.	Year of Release	Place of Origin (US)	PI or CI Number	Rht-B1/Rht-D1 ^b^
Turkey	1870	Landrace	CItr 5757	a/a
Kharkof	1900	Landrace	PI 5641	a/a
Cheyenne	1933	UNL	CItr 8885	a/a
Red Chief	1940	Kansas	CItr 12109	a/a
Wichita	1944	KSU	CItr 11952	a/a
Warrior	1960	UNL	CItr 13190	a/a
Lancer	1963	USDA/UNL	CItr 13547	a/a
Triumph 64	1964	OSU	CItr 12132	a/a
Sturdy	1966	TAMU	CItr 13684	b/a
Scout 66	1967	UNL	CItr 13996	a/a
Clark’s Cream	1972	Kansas	PI 476305	a/a
Centurk 78	1978	UNL	CItr 17724	a/a
Centura	1983	UNL	PI 476974	a/a
Siouxland	1984	UNL	PI 483469	a/a
TAM 107	1984	TAMU	PI 495594	b/a
Wesley	1998	USDA/UNL	PI 605742	b/a
Jagalene	2002	Monsanto	PI 631376	b/a
Anton	2007	USDA	PI 651044	b/a
Overland	2007	UNL	PI 647959	b/a
Camelot	2008	UNL	PI 653832	b/a
Settler CL	2008	UNL	PI 653833	b/a
Mattern	2012	USDA/UNL	PI 665947	b/a
Freeman	2013	UNL	PI 667038	b/a

^a^ USDA, US Department of Agriculture, UNL, University of Nebraska-Lincoln; TAMU, Texas A&M University; OSU, Oklahoma State University; KSU, Kansas State University; PI or CI obtained from the USDA-Agricultural Research Service National Plant Germplasm System Database: https://npgsweb.ars-grin.gov/gringlobal/search.aspx (accessed on 14 September 2022). ^b^ Rht-B1/Rht-D1 alleles are ‘a’ tall and ‘b’ semi-dwarf [24,25,26].

### 2.2. Kernel Quality

Kernel physical characteristics were recorded using a single kernel characterization system (SKCS4100, Perten, Stockholm, Sweden). Kernel hardness index (HI), hardness distribution, kernel moisture, kernel diameter, kernel weight, and their standard deviations were recorded following the manufacturer’s instructions. HI is calculated based on the force required to crush each kernel. It is measured on an arbitrary scale where hard kernels have a HI > 65, semi-hard 45 < HI < 64, semi-soft 35 < HI < 44, and soft kernels HI < 25. Since kernels are measured individually in the SKCS, the standard deviation of each parameter is a useful value that gives a measure of the consistency in kernel texture and dimensions.

Kernels were tempered and milled using a Quadrumat Jr laboratory mill [27,28]. All samples were tempered at 15% moisture content overnight before milling. Flour and bran were separated using a no. 70 sieve with 212 µm openings. Milling yield was calculated as the weight of flour recovered divided by the weight of the starting wheat.

### 2.3. Flour Quality

Moisture content of flour was determined following an approved method [29]. Flour protein concentration was measured by a nitrogen analyzer (FP 528, Leco, St. Joseph, MI, USA) with a nitrogen conversion factor of 5.7 [30].

Flour mixing quality was assessed using a Mixograph (National, Lincoln, NE, USA) [31]. Midline peak time (MPT), midline peak value (MPV), midline peak width (MPW), midline right slope (MRS), and midline time max area (MTA) were recorded from the Mixograph to evaluate the mixing quality of white flour.

### 2.4. Baking Quality

Bread was baked according to an approved straight-dough method using 30 g of flour and a fermentation time of 90 min [32]. The mixing time and water absorption of flours were determined from the Mixograph results. Baked bread was cooled to room temperature for 1–4 h before further testing.

Loaf volume and specific volume (cm^3^/g) were determined by the rapeseed displacement method [33]. Firmness was obtained using a texture analyzer equipped with a 1 cm cylindrical probe and a 2 kg load cell (TA-XT2, New York, NY, USA) [34].

### 2.5. Bran Quality

Friability of bran was measured by sieving the bran obtained after milling through two testing sieves stacked on top of each other for 60 s (no. 20 and no. 60 containing 850 µm and 250 µm openings, respectively) [17]. *Friability* was calculated according to the following equation:

(1) $$Friability\;\left(\%\right)=\left[W_{60}/(W_{60}+W_{20}\right)]\times 100\%$$

where $W_{60}$ was the weight fraction of fine bran remaining on sieve No. 60 (fine bran particles), $W_{20}$ was the weight fraction of fine bran remaining on sieve No. 20 (coarse bran particles).

Water retention capacity of bran was obtained as described by Navrotskyi et al. [17]. In short, 1 g of bran was mixed with 5 mL of water. After vortex mixing for 5 s, samples were shaken on a horizontal shaking platform at room temperature and 100 rpm for 20 min. Then, samples were centrifuged at 1000× *g* for 15 min, and the supernatant was discarded. After draining the pellet upside down over paper towels for 10 min, the weight of the wet pellet was recorded. BWRC was calculated as the ratio of the weight of the wet pellet to the weight of dry bran, expressed as a percentage.

### 2.6. Statistical Analysis

Data were analyzed using a mixed model ANOVA. The main effects were release year of cultivars and harvest year. Release year was modeled as a continuous variable, and harvest year was a fixed variable. Replication nested within planting year was a random effect. Because historical and modern cultivars differ in height due to the presence of dwarfing genes (Table 1), plant height was included in the ANOVA model as a co-variate to address differences in plant stature. Statistical significance was determined by *p* < 0.05.

Partial correlation using harvest year and plant height as the partial variables was used to determine the relationships among variables. All statistics were performed using SAS software (version 9.4, Cary, NC, USA). Data were plotted using the ‘ggplot2′, ‘cowplot’, and ‘corrplot’ packages in R (version 4.0.3) [35,36,37].

## 3. Results

### 3.1. Kernel Quality

**Figure 2:**
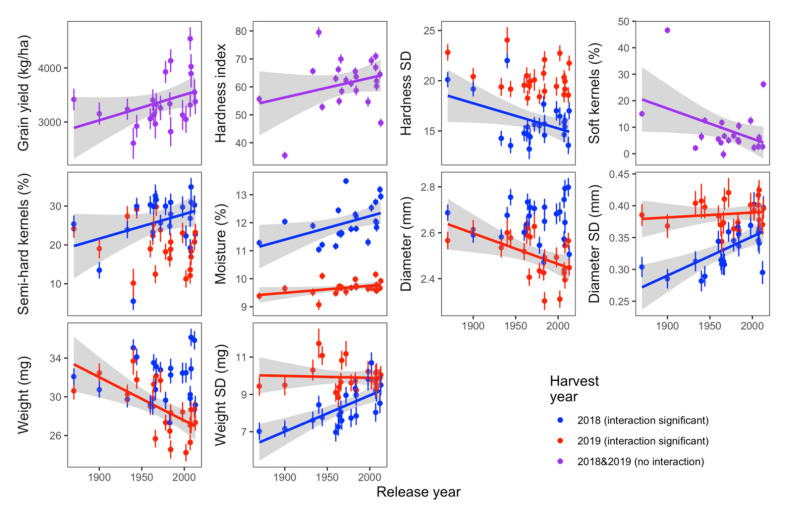
Kernel characteristics and grain yield with significant trends across release year. For variables with a significant release year × harvest year interaction, least squares means are plotted by harvest year. For variables where only the main effect of release year was significant, least squares means are plotted across both harvest years. Regression lines are plotted only for data with a significant trend across release year. The gray shaded area shows the 95% confidence interval of the regression line.

Grain yield was significantly increased with release year (Figure 2). Kernel physical characteristics included the mean and standard deviation of kernel HI, moisture content, diameter, weight, and hardness distribution. Kernel texture (HI, % soft kernels, and % semi-hard kernels) varied by release year with no interaction with harvest year and interaction, respectively (Table 2). Scatterplots of the least-squares means of these variables indicated that kernels from modern cultivars tended to be harder (Figure 2).

Kernel moisture and dimensions (diameter and weight and standard deviation of diameter and weight) varied as a function of the interaction between release year and harvest year. A plot of the data showed that kernel diameter and weight decreased with release year in 2019, with no trend in 2018 (Figure 2). In 2019, there was much less rain during grain filling (June; Figure 1). Therefore, it appeared that modern wheats were more affected by lack of rain during grain filling compared with historical wheats manifesting in smaller kernels. The trends for kernel diameter and weight standard deviations revealed that kernels have become less uniform in size and weights across release years (Figure 2). Overall, there was an increasing trend in kernel moisture content (Figure 2). Given the transient nature of moisture content, it was surprising that significant trends existed across release years for both harvest years, considering that samples were produced, harvested, and stored under the same conditions within harvest year and analyzed in random order. No trend was found between milling yield with release year in this population (Table 2).

### 3.2. Protein Quality

**Figure 3:**
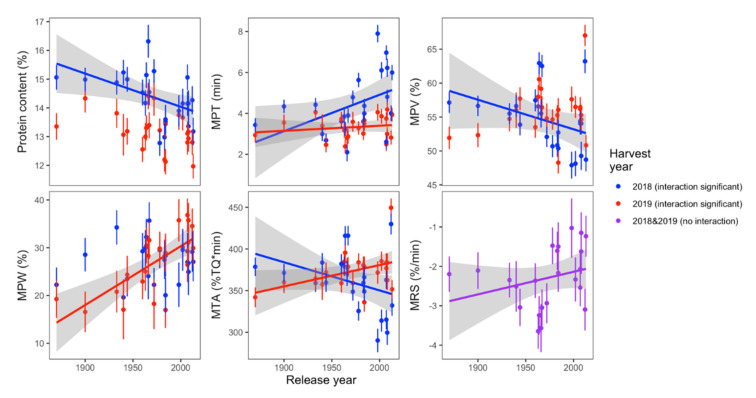
Protein content and mixing quality with significant trends across release year. For variables with a significant release year × harvest year interaction, least squares means are plotted by harvest year. For variables where only the main effect of release year was significant, least squares means are plotted across both harvest years. Regression lines are plotted only for data with a significant trend across release year; the gray shaded area shows the 95% confidence interval of the regression line. (MPT: Midline peak time; MPV: Midline peak value; MPW: Midline peak width; MTA: Midline time max area; MRS: Midline right slope).

The flour protein concentration varied as a function of the interaction between release year and harvest year (Table 2). Analysis by year indicated a strong decrease in protein concentration in the 2018 harvest year that was not evident in 2019 (Figure 3). Flour mixing quality variables also varied with release year interacting with harvest year. Midline peak time (MPT) and dough tolerance to overmixing (MRS) had significant increasing relationships with release year in both harvest year. MTA had a crossover effect in two harvest years (Figure 3).

No significant relationship was found between bread volume, specific volume, and firmness with release year (Table 2). Notably, even though flour protein content decreased with release year, there was no decreasing trend between baking quality and release year (Table 2 and Figure 3).

### 3.3. Bran Quality

**Figure 4:**
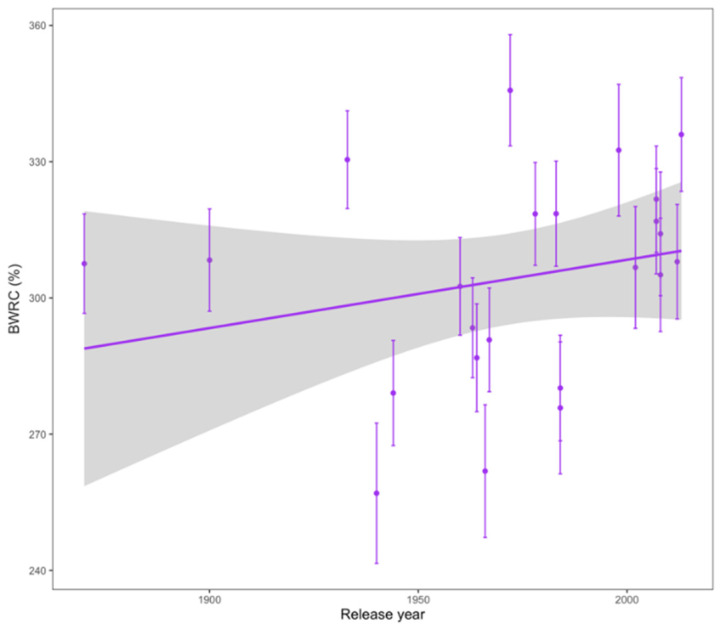
Bran water retention capacity (BWRC) with significant trends across release year. Least squares means are plotted across both harvest years. The gray shaded area shows the 95% confidence interval of the regression line.

In this study, we evaluated two bran quality traits that are related to whole wheat bread baking quality: BWRC and bran friability. No trend was found between bran friability and release year. However, BWRC had a significant increasing relationship with release year (Table 2 and Figure 4).

### 3.4. Correlation among Quality Parameters

**Figure 5:**
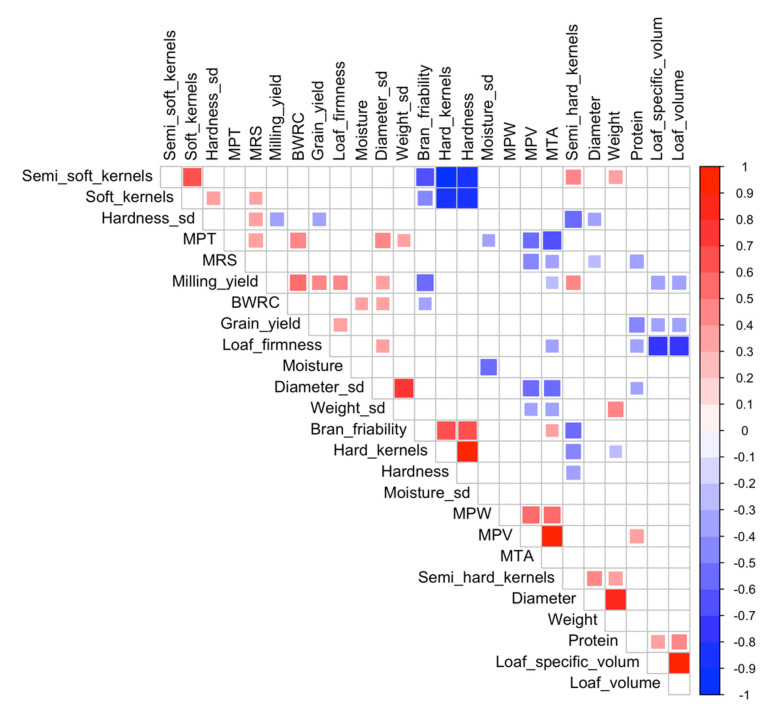
Partial correlation between each variable. Partial variable was harvest year, and plant height. Least-squares means in each harvest year were used in the correlation (*n* = 46). Only significant correlations are plotted (*p* < 0.05). (MPW: Midline peak width; MPV: Midline peak value; MTA: Midline time max area; MPT: Midline peak time; MRS: Midline right slope; BWRC: Bran water retention capacity).

Correlations among each variable are shown in Figure 5. Several expected correlations were observed between hard and soft kernels and kernel dimensions. Apart from these, grain yield has negative relationship with kernel hardness uniformity, protein content, and loaf-specific volume and volume. It is positively correlated with milling yield and loaf firmness. Moisture content and kernel diameter uniformity were positively correlated with BWRC. Bran friability had a positive correlation with kernel hardness and MTA and a negative correlation with milling yield and BWRC. Loaf-specific volume had a strong positive correlation with loaf volume. Both loaf-specific volume and volume had significant positive relationship with protein content and negative relationships with loaf firmness. Loaf firmness is negatively related with protein content.

## 4. Discussion

As little information exists on the changes in end-use quality between historical and modern winter wheats adapted to the Great Plains of the US, we examined 23 representative cultivars of wheat released between 1870 and 2013 that were widely grown in this region during their time. We analyzed the changes in end-use quality and examined these changes in the context of similar studies discussing changes in other classes of wheat or adapted regions.

Kernel hardness increased with release year. Similar results were reported in another study with spring wheat over time [4]. Kernel hardness is an important milling parameter that should be measured before milling. Kernel hardness is significant to the milling process because kernel texture can impact power consumption during milling, with harder kernels requiring more energy [13]. Hard kernels also require a longer tempering time and need to reach a higher moisture content before milling than soft kernels. Harder wheat is more difficult to break down and may produce larger particles after milling [38]. The variability in kernel hardness also decreased with release year. Kernel hardness uniformity is desirable in the milling industry because tempering and milling conditions do not have to be varied as much to obtain optimum flour yield [14,15].

We found a significant positive relationship between kernel moisture content and release year. Moisture content is a transient property of wheat kernels that changes depending on humidity and whether the kernels are gaining or losing moisture. Therefore, given that all the kernels were produced, harvested, stored, and analyzed under the same conditions in random order, it was unexpected that a significant relationship existed between moisture content and release year. Although all samples were below the moisture content required for microbial growth, the apparent tendency of modern cultivars to equilibrate to elevated moisture contents may be a food safety concern. In recent years, there have been an increased number of microbial food safety issues caused by wheat flour [39,40]. The reasons why modern cultivars seem to equilibrate to higher moisture contents and the relationship to survival of pathogens and other food safety concerns may merit further investigation.

The decrease in kernel dimensions (diameter and weight) is in contrast to a previous study where kernel diameter was shown to increase with release year [4]. This could reflect differences in priorities among breeding programs in terms of quality. It has been reported that smaller kernels can have lower flour yield than large kernels because they have lower proportion of endosperm relative to bran [10]. However, smaller kernels can have better bread baking quality in terms of loaf volume and Mixograph peak time than larger kernels [10]. The decrease in kernel size uniformity across release year is not desirable. Kernel size uniformity has many effects on wheat milling. It can affect the flour yield, ash content, and the grinding process. High kernel variability also causes higher attrition to the milling machine [13]. Although no trend was found between milling yield with release year in this population, another study reported that milling yield has a positive correlation with release year due to the increase in the kernel size over time with spring wheat [4]. It has been reported that larger kernels can have higher flour yield and friability and lower endosperm separation index [10,41,42]. In our study, the correlations between kernel size and milling quality are not very high when using harvest year as the partial variable. Because we did not observe a significant trend between size and release year, it is reasonable to not have a significant relationship between milling yield and release year. Brorsen et al., also found kernel diameter is significantly correlated with MPT [43].

The decrease in protein concentration in modern wheat compared with historical wheat that was reported in this study has been reported in many previous studies [6,44,45]. Overall, the trends associated with mixing time (MPT) and dough tolerance to overmixing (MRS) showed improvements with release year. Thus, protein mixing quality increased even while total protein decreased. The MPT is the time when the dough has optimum elasticity. Generally, a longer MPT is desirable to allow for adequate mixing of ingredients into the dough before it is developed. The longer the peak time (MPT) and lower midline right slope (MRS) means the dough has a higher tolerance to overmixing. In our study, MPT increased and MRS decreased with release year, which means the dough elasticity and tolerance increased during breeding. Peak height (MPV) is indicative of dough strength, peak widths (MPW) are indicative of mixing tolerance, and peak areas are indicative of dough consistency. Small negative slope values indicate a flatter curve, which is preferable to large negative slope values, indicating poor tolerance to mixing. Peak height is reached when optimum hydration has occurred. Therefore, peak height is a function of protein content and water absorption [46]. The improvement of mixing quality in terms of longer development time and better tolerance has been reported in many studies [4,20,22].

No relationship was found between release year and bread volume, specific volume, and firmness in the present study. This is in accordance with a previous study that showed no correlation between loaf volume and release year, although the previous study did report a negative correlation between loaf firmness and release year [4]. Thus, although the physical characteristics, milling quality, mixing quality of wheat kernel results have shown changes over time, their impact was not enough to influence overall baking quality. Additionally, because flour protein content decreased with release year, the protein in modern wheat is more functional in terms of having better elasticity and pseudoplastic behavior. This is reflected in the improvements in some of the mixing quality parameters. The reason we did not observe improvements in baking quality may be because breeding programs typically evaluate kernel characteristics, milling, and mixing quality in early generation lines, but the baking quality is usually only evaluated in late generation lines because it uses so much more flour and is so much more time consuming [47]. A previous study reported that grain hardness, flour water absorption, and whole wheat bread volume were strongly associated [48].

BWRC increased across release year. Because BWRC is inversely related to whole wheat bread quality [17], the increasing BWRC across release year suggests a decrease in whole wheat bread quality over time. Bran friability was not related to release year but was correlated with BWRC. This suggests that bran friability may be a positive characteristic of whole wheat flours although very fine bran particles tend to decrease bread quality [16,49]. Higher bran friability also makes separating flour from bran more difficult and can reduce refined flour milling yield [50]. With the emphasis on increasing whole grain consumption, it is important to include whole grain quality parameters in breeding decisions in order to maintain or improve the quality of whole grain foods.

## 5. Conclusions

In the present study on winter wheat adapted to the Great Plains of the US, we found that along with increased grain yield, wheat kernels have become harder and more variable in shape over nearly a century of wheat breeding. Concurrently, the flour protein has decreased, yet mixing quality has improved, and baking quality has been maintained, indicating important improvements in protein functionality.

This study also revealed potentially important areas for future research. First, the tendency for modern wheat kernels to equilibrate to higher moisture contents may be a food safety concern worthy of investigation. Second, the increase in BWRC suggests that whole wheat bread quality may be decreasing over time, which has not been previously reported and may be important given the emphasis on whole grain consumption by nutritionists and government agencies.

## Figures and Tables

**Table 2:** ANOVA (mean squares) among kernel physical characteristics and release year, harvest year, and their interaction with plant height as a co-variate.

Variable	HY	RY	Height	HY × RY
Grain yield	151.42 ***	5.05 *	5.94 *	3.28
Hardness index	36.46 ***	6.07 *	6.99 **	1.54
% soft kernel	4.76 *	21.02 ***	6.13 *	0.16
% semi-soft kernel	28.86 ***	0.04	3.90	3.93 *
% semi-hard kernel	41.39 ***	4.17 *	0.04	4.57 *
% hard kernel	34.43 ***	3.17	4.97 *	2.04
Hardness std	156.29 ***	8.99 **	6.02 *	8.09 **
Moisture (%)	803.40 ***	17.43 ***	0.86	5.49 *
Moisture std (%)	1143.37 ***	0.04	2.07	5.56 *
Diameter (mm)	134.47 ***	4.57 *	25.09 ***	12.19 ***
Diameter std (mm)	114.72 ***	44.75 ***	1.43	14.88 ***
Weight (mg)	100.14 ***	4.95 *	20.95 ***	14.06 ***
Weight std (mg)	77.14 ***	49.84 ***	0.82	10.52 **
Milling yield (%)	10.06 **	0.18	0.53	1.60
Bran friability (%)	63.22 ***	0.46	2.39	2.50
BWRC (%)	160.53 ***	9.35 **	3.35	0.03
Protein content (%)	72.78 ***	18.56 ***	0.75	3.99 *
MPT (min)	58.45 ***	29.11 ***	7.51 **	10.23 **
MPV (%)	4.14 *	1.79	1.34	8.19 **
MPW (%)	4.88 *	4.09 *	0.01	5.83 *
MRS (%/min)	10.07 **	9.04 **	11.65 ***	0.96
MTA (%TQ·min)	7.25 **	0.77	2.14	12.48 ***
Loaf Volume (cm^3^)	1.12	0.01	5.77 *	0.26
Loaf specific volume (cm^3^/g)	2.02	0.53	7.77 **	0.00
Texture (g)	0.18	0.34	6.36 *	0.93

* *p* < 0.05; ** *p* < 0.01; *** *p* < 0.001.

## Data Availability

Data is contained within the article and its Supplementary Materials.

## Supplementary Materials

The following supporting information can be downloaded at: https://www.mdpi.com/article/10.3390/foods11192975/s1, Table S1: Raw data for response variables across two planting years (2018–2019) and twenty-three cultivars.
